# All things to all people: trade-offs in pursuit of an ideal modeling tool for maternal and child health

**DOI:** 10.1186/s12889-017-4751-4

**Published:** 2017-11-07

**Authors:** Timothy Roberton, Kate Litvin, Andrew Self, Angela R. Stegmuller

**Affiliations:** Department of International Health, Johns Hopkins Bloomberg School of Public Health, Baltimore, MD USA

## Abstract

**Background:**

Modeling tools have potential to aid decision making for program planning and evaluation at all levels, but are still largely the domain of technical experts, consultants, and global-level staff. One model that can improve decision making for maternal and child health is the Lives Saved Tool (LiST). We examined respondents’ perceptions of LiST’s strengths and weaknesses, to identify ways in which LiST – and similar modeling tools – can adapt to be more accessible and helpful to policy makers.

**Methods:**

We interviewed 21 purposefully sampled LiST users. First, we identified the characteristics that respondents explicitly stated, or implicitly implied, were important in a modeling tool, and then used these results to create a framework for reviewing a modeling tool. Second, we used this framework to categorize the strengths and weaknesses of LiST that respondents articulated.

**Results:**

Two overarching qualities were important to respondents: usability and accuracy. For some users, LiST already meets these criteria: it allows for customized input parameters to increase specificity; the interface is intuitive; the assumptions and calculations are scientifically sound; and the standard metric of “additional lives saved” is understood and comparable across settings. Other respondents had different views, although their complaints were typically not that the tool is unusable or inaccurate, but that aspects of the tool could be better explained or easier to understand.

**Conclusion:**

Government and agency staff at all levels should be empowered to use the data available to them, including the use of models to make full use of these data. For this, we need tools that meet a threshold of both accuracy, so results clarify rather than mislead, and usability, so tools can be used readily and widely, not just by select experts. With these ideals in mind, there are ways in which LiST might continue to be improved or adapted to further advance its uptake and impact.

**Electronic supplementary material:**

The online version of this article (10.1186/s12889-017-4751-4) contains supplementary material, which is available to authorized users.

## Background

High on the international health agenda is the need to make better use of data for decision making [1]. There is widespread appreciation of the value of health program data, and increasing expertise in collecting them, but comparatively limited tools and guidance on how data should be used, particularly at district and regional levels. Many collected data are compiled into national or global aggregates without being used at lower levels. Other data are neglected because they are incomplete or seemingly unhelpful in their raw state, and have not been transformed into useful information. For effective resource allocation and program implementation, we need better support for data use at all levels.

Mathematical modeling is one technique that can enable more effective data use, by extrapolating from given data to produce estimates that would not otherwise be available [2]. In the field of maternal, newborn, and child health (MNCH), models have been developed to understand time trends in child and maternal mortality [3], budget the cost of new policies [4], estimate the impact of scaling-up vaccine coverage [5], and generate equity-disaggregated outcome measures [6]. Policy makers who use these tools have more robust evidence with which to develop policies and set priorities than policy makers who draw on raw data alone.

Although the use of modeling tools in MNCH is gaining traction, they are still largely the domain of trained specialists, consultants, or global-level staff. There are few examples of mid-level government or NGO staff using models independently to inform regional- or district-level decisions. Public health practitioners may want modeled information, but lack the time or skills to run models, and instead outsource modeling to other groups. Many organizations could see gains in capacity, efficiency, and improved decision making if workers were able to use modeling tools themselves.

LiST is an example of a modeling tool that aims to improve MNCH decision making. With LiST, users can estimate how increased coverage of interventions will reduce deaths due to common maternal and child diseases. Users can model scenarios to determine which interventions will contribute most to reductions in mortality, measure the impact of programs in terms of “lives saved”, and combine estimates with costing data to understand the cost-effectiveness of proposals.

The LiST team at Johns Hopkins University wants LiST to be used by policy makers as extensively as possible. In this paper we explore the barriers and opportunities for further uptake of LiST. We use qualitative data from semi-structured interviews with LiST users to examine the model’s strengths and weaknesses – both conceptually, in terms of the mathematical model and the information it produces, and practically, in terms of its usability as a software package. We end by suggesting ways in which LiST and similar modeling tools can adapt to be yet more accessible and helpful to policy makers.

## Methods

We collected qualitative data from LiST users through semi-structured interviews. We purposefully sampled respondents from the LiST mailing list and from a shortlist of researchers and practitioners not on the mailing list but known by LiST team members to have used LiST in recent years. The study team categorized potential respondents by organization and role, and selected 26 people who: (i) either used LiST themselves or had commissioned LiST analyses from others and used the resulting projections; and (ii) reflected a range of organizations (government agencies, NGOs, academic institutions), roles (policy makers, managers, technical staff, researchers), geography (high- and low-income countries), and use cases (evaluation, priority setting, program planning). The 26 sampled LiST users were invited by email to participate in the study: 22 replied to the invitation after one or two email attempts; of these, 1 declined to participate and 21 were interviewed.

A semi-structured interview guide was used by two interviewers trained in qualitative methods, who conducted interviews in person (*n* = 2) and by phone (*n* = 19). Both of the interviewers (KL and AS) and the study coordinator (TR) were involved in developing the study protocol and creating the interview guide, which we believe led to high inter-interviewer reliability. Questions focused on respondent’s experiences of LiST, the nature of their work using LiST, and their perceptions of its strengths and weaknesses as a policy-making tool and software package. Respondents were interviewed only once, with no follow-up. No remuneration was offered to respondents. All interviews were audio recorded. Interviewers took notes during interviews and used audio recordings to verify data and quotations. Throughout data collection, interviewers met as a team with the study coordinator to review findings, to ensure that questions were being asked in the same manner by each interviewer, and to identify themes for greater focus. The interview guide is available in Additional File 1.

We conducted data analysis in two phases. In Phase 1, to more fruitfully organize and compare respondents’ comments, the study team developed a heuristic of the stages or processes involved in using a model such as LiST. These processes were: (i) gathering and inputting data, (ii) running the model, and (iii) outputting and interpreting results. For each of these processes, we examined our interview data to identify the qualities that respondents explicitly stated, or implicitly suggested, were important in a modeling tool. We then used these process and quality dimensions to create a framework for assessing the strengths and weaknesses of a modeling tool. In Phase 2, we applied this framework to LiST by grouping respondents’ comments about the model’s perceived strengths and weaknesses, and their suggestions for improving the model, according to the cells of the framework.

## Results

### Building the analytical framework

As respondents talked about each of the processes involved in using LiST (gathering and inputting data, running the model, and outputting and interpreting results), respondents repeatedly identified two qualities that were important in a modelling tool: usability and accuracy.

#### Usability

A common concern was how easy or difficult LiST is to use. Respondents reported that a good tool is “user friendly”, easy to learn, with minimal barriers to conducting analyses. Many respondents highlighted the potential for tools such as LiST to be used by colleagues in low- and middle-income countries (LMICs) to assist local target setting. Given the often high turnover at national and district health offices, respondents said that staff need to be able to use a tool with only minimal experience or training.

*“There is a big disconnect between global level strategic think tanks that run international organizations, NGOs, where they sit and are familiar with evidence, research, methods, and have good access to all of the information. ... We don't want to further this situation where there is always a need for assistance. We really want to make knowledge and skill acquisition accessible to colleagues who have not had access to it and use it in their specific context.”*

*“In terms of public health, having a tool that can be simple enough to be able to be used with minimal training or with the training that is available online, but yet can be adapted to local purposes, I think is really important.”*

#### Accuracy

The other overarching quality that was important to respondents was accuracy. Respondents frequently mentioned the importance of LiST producing valid results. LiST may be easy to use, but this means little if its assumptions and outputs are not based on scientific evidence or appropriate to a user’s scenario. Respondents who said that LiST was evidenced-based and scientifically rigorous typically cited these characteristics as its main strength.

*“[Modeling tools] don’t replace country discussions about priorities, but they can help facilitate a discussion about priorities by putting a layer of evidence and objectivity to that discussion.”*

*“LiST is state of art in terms of the answers it gives. Nothing else will give you any better answer than LiST does. It’s kept up to date and it’s evidence based, tells you calculations it’s doing in the background, in terms of what current coverages are and in terms of effect sizes.”*

Using these two qualities of usability and accuracy, and the three processes involved in creating a model, we built a framework for assessing the strengths and weaknesses of a modeling tool. Table 1 shows this framework with examples of the characteristics that respondents in our study said they valued.

**Table 1:** Framework for assessing a modeling tool’s strengths and weaknesses, with examples of the characteristics that respondents in our study said they valued

Model qualities	Model processes		
	Gathering and inputting data	Running the model	Outputting and interpreting results
Usability	▪ Required input data are feasible to gather ▪ Default data are available within the tool	▪ Interface is clear and logical ▪ Software is accessible with minimal operating requirements ▪ Time needed to run model is minimal	▪ Results are well-explained and easily interpretable ▪ Output metrics reflect standard indicators that are meaningful to target audiences ▪ Output products (files, data, images) are available in appropriate formats
Accuracy	▪ Sufficient input options are available for specifying scenarios ▪ Default data are accurate	▪ Assumptions are plausible ▪ Model calculations are correctly executed	▪ Results are sufficiently detailed ▪ Results are appropriate to user’s analysis questions

### Using the framework to analyze LiST

We used the framework in Table 1 to organize respondents’ comments on LiST. Below we describe the reported perceptions of LiST’s strengths and weaknesses at each stage of the modeling process.

#### Gathering and inputting data

When users open LiST they have the option to customize all inputs. Although this flexibility was attractive to some respondents, to others it was daunting. Several users said they appreciated the rigor that LiST offers in its assumptions and customizable input values, but others said they found it challenging to gather the needed data and confusing as to how to enter data correctly. Inputting data was more challenging for certain types of analysis, such as subnational projections.

*“I think it will be really helpful to further develop the guidance for conducting these types of analyses at the subnational level. Having clarity and guidance about the type of data that are needed.”*

Many respondents made the point that a LiST projection is only as valid as the data used to create it. Respondents stressed the importance of up-to-date inputs for coverage and effectiveness of interventions, cause-of-death distributions, and baseline mortality data.

*“The problem is that LiST is only as strong as the data that it has, and data is hard to come by at the best of times and otherwise we rely on five-year intervals for DHS data, and that can be misleading sometimes or have gaps.”*

*“Also a big challenge that we have is the quality of data that we’re using.”*

Some users said that it was up to the user to provide their own input data. Others expected these data to be available in LiST by default. LiST currently comes with default health status data, intervention effectiveness values, and coverage data, drawn from DHS, MICS, and other surveys [7]. The fact that these defaults are *built in* increases the tool’s usability. The *validity* of the defaults, and their appropriateness for the scenario the user wants to model, increases the tool’s accuracy. Currently LiST only has default data for national-level projections.

*“LiST really does provide a fantastic vessel... It uploads DHS data and other information from the source, which helps consolidate information. It is quite complex with so many factors that have sophisticated ways of interacting with each other. It helps to have a lot of the math and number crunching being done by the software.”*

#### Running the model

Respondents had mixed thoughts on the usability of the LiST interface for running models. Most seemed to be happy with the interface, including one user who said it was “intuitive”, but a few respondents said they found it challenging to manipulate.

*“We’ve actually created a few Excel based modeling tools to try to do the same thing, actually that is one of the nice things about LiST is the standardization of the software to produce those estimates. Rather than different teams using excel programs with different assumptions and different inputs available to them.”*

*“I have seen [user interface] progress drastically in the past seven years, when I think about what we had originally and what we have now, it’s incredible. I really would commend the team on the progress they’ve made. I think features like when you hover over data and its gives the definition or the source of the data, that kind of information is really helpful.”*

Despite their appreciation for the interface, many respondents requested more support and guidance on how to run models, including more help features in the software (for example, warnings when improbable estimates or targets are entered). The LiST team currently offers in-person trainings and online tutorials for users to teach themselves how to conduct simple analyses. Some respondents mentioned and appreciated these tutorials, but others said they were insufficient for more complicated projections. Users also requested greater clarity on how LiST’s assumptions and calculations change from version to version.

For many respondents, a key strength of LiST was its mathematical approach and the algorithm it uses to calculate results. Some users said the quantitative evidence built into LiST – for example, the default effectiveness values – gives them greater confidence in using the results for advocacy purposes.

*“The fact that it’s being used in other areas, and people are somewhat familiar with it… When we present information from LiST, it’s not like it’s some unproven tool that we’re using. And it is getting better traction. It is a model that people would agree on, or at least that it is on the right track.”*

*“There is not really a better way to make systematic evidence-based decisions.”*

*“There are some equally clunky pieces of software, like [program X], but [they’re] based on really shaky assumptions. Not only [is program X] not user friendly, but also I’m really skeptical of any answer it comes up with. Doesn’t have a good evidence base and it’s for advocacy, which drives me crazy.”*

*“Even with all that LiST has produced, in terms of academic papers, to justify the estimates that are used, you are always going to have naysayers who question the reliability of models and concerned with using them.”*

Meanwhile, other users questioned LiST’s assumptions, or their *understanding* of LiST’s assumptions, and suggested that these needed to be better documented. A few respondents said they found the calculations for some aspects of LiST confusing or potentially misleading.

*“I assumed what LiST had in there for wasting was really solidly planned and based on the best reviews out there. But, out of my own curiosity, I started digging in a bit further and read the systematic review the effect size was based on. … From my reading of the systematic review that was the basis for what is in LiST, I would not have recommended the intervention at all, or definitely not the effect size. In that review the authors were very cautious and said the results were kind of inconclusive, whereas in LiST it didn’t mention that and just had an effect size and referenced this systematic review.”*

Most of the users who had negative perceptions of LiST’s accuracy were not concerned that the model itself is inaccurate or wrong, but that they, and others, might not have the skills or information to tell if their projections are accurate or were produced correctly. One respondent said that because their team does not understand how LiST’s results are generated, their organization is hesitant to use LiST for program planning.

*“Sure they can read a couple of articles published and run the results, but that is going to have kind of negative implications, if they do it wrong.”*

*“There is still some concern about the model and the estimates that come out of LiST. … I know [the LiST team] has tried very hard to be transparent in the effect sizes that have gone in there and what assumptions are made around LiST. But I think [they need to] increase transparency and make people aware of all the work that has gone into LiST – that it isn’t some magic black box. Increasing people’s awareness will be useful.”*

#### Outputting and interpreting results

Several respondents appreciated the standard metric of “additional lives saved” that LiST gives; that it is easy to understand, and easily comparable across programs, interventions, and country settings. A recurring comment was the value of LiST as a tool for quantifying and communicating the potential impact of programs for advocacy purposes. Respondents noted the importance of providing clear, meaningful data to decision makers. Users said that a strength of LiST is that one does not need background knowledge to understand the significance of its results.

*“For communication, it’s often difficult to communicate what the impact of coverage changes are in terms of health outcomes. That’s what LiST has been most useful for, is the external communication.”*

*“Having one tool that can compare across different settings is very useful, as standardization allows for comparisons.”*

Despite this, some users expressed concern about the potential for colleagues or inexperienced users to misinterpret LiST’s results, or to not account for the assumptions it makes or the quality of input data.

*“The interpretation of LiST does not allow you to understand the differences and nowhere have I ever seen it clearly laid out in any of the literature.”*

*“I think that at times there’s perhaps a lot of unfair scrutiny because of certain projects/studies or papers that have used LiST to make wider conclusions than should have been drawn, but I don’t that that is the fault of the tool, it doesn’t mean the tool is wrong. I think the uses can be more or less appropriate.”*

Respondents mentioned other outputs, unavailable in LiST, which would be valuable in future versions. One respondent suggested that results could be automatically disaggregated by income level, gender, or other subgroups. Another user suggested that LiST could consider the effects of non-communicable diseases on mortality and morbidity. Another request was to account for the *quality* of intervention coverage, with results that more highlight the impact of poor- versus high-quality services. Finally, one respondent asked to incorporate *optimization* into LiST: for example, having LiST automatically identify the most efficient combination of coverage increases to achieve a given mortality reduction.

## Discussion

It seems that for a modeling tool to serve the needs of policy makers at global, national, and local levels, it must be both easy-to-use and accurate. At least for some users, LiST already meets these criteria: it allows for customized input parameters to increase specificity; the interface is intuitive; the assumptions and calculations are scientifically sound; and the standard metric of “additional lives saved” is understood and comparable across settings. Some respondents had different views, though their complaints were not that the tool is completely unusable or inaccurate, but that aspects of the tool are not well explained and are difficult to understand.

These findings are limited by our sampling strategy and the fact that we only interviewed people who we knew had already used LiST. A study that sought to more rigorously assess perceptions of LiST’s strengths and weaknesses might randomly sample users from a wider pool of MNCH experts, or take a sample of non-users, train them on LiST, and test their ability to use it. But even with a non-random sample, our study revealed various challenges to increasing the uptake of LiST. Arguably those who have used LiST regularly are more attuned to its limitations.

### The usability-accuracy trade-off

One frequent theme in our data was that the characteristics of LiST that were identified as weaknesses were often related to the characteristics identified as strengths. For example, some respondents were overwhelmed by LiST’s many input parameters, but others valued the wide array of options for customizability. Some appreciated that “additional lives saved” was an easy metric to interpret, while others found this simplistic and open to misinterpretation. Ease-of-use appealed to one user, but over-simplification was limiting to another.

This highlights something of a trade-off between usability and accuracy, with increased accuracy *necessarily* making the tool more complicated to use. Consider, for example, the need for input data. A completely user-friendly tool might work straight “out of the box”, with limited user attention needed to fine-tune input parameters. The user would not have to set custom coverage data or effectiveness values, but could instead draw on default data. But by necessity, these default values would be more “generic” than if the user had set them themselves, and because the values would be generic, the results would be less specific, or accurate, to the user’s context.

Consider also the use of “single indicator” inputs. Currently in LiST, instead of entering detailed, age-specific data on stunting, users can enter a single value and LiST will make assumptions about how this value disaggregates to age-specific categories. For childbirth interventions, a single indicator of institutional delivery will be separated by LiST into intervention-specific coverage values using a default algorithm [8]. In both cases, taking the less demanding route of using a “single indicator”, increases LiST’s reliance on default assumptions. Greater precision requires the user to enter more data.

So there is a conundrum. A minimalist interface that relies on defaults runs the risk of inappropriate models that do not capture the specifics of a scenario, and opens the door to misinterpretation by inexperienced or time-pressed users. But on the other hand, increased demands on the user will seemingly limit the tool’s uptake. If we want a modeling tool to be used by more policy makers – a tool that users will trust but is accurate enough to meet their demands – what is the way forward?

### The way forward

The following ideas represent improvements or adaptations that might be made to LiST to increase either usability or accuracy for certain types of users, or both where possible. Some of these ideas were recommended by respondents in our study; others reflect our own suggestions. Many of these recommendations are currently being explored by the LiST team and are anticipated to be incorporated into future versions of the tool.

#### Tolerating compromises in accuracy where appropriate

First, we can think critically about what level of accuracy is required of a model, and what compromises might be acceptable. Models are inherently inaccurate: assumptions are limiting, and available data rarely matches the known need for data. For particular tasks and decisions, some uncertainty may be acceptable. However, in reducing complexity there are choices to be made, and reducing complexity in some areas may be more acceptable than in others. For example, a model could prioritize influential parameters over others, forcing users to find inputs that will have more influence on outputs, and relegating to generic data those parameters that have less influence. This requires an understanding of the mathematical implications of adjusting each parameter, balanced by the difficulty of gathering and inputting the data.

#### Reducing unnecessary interface complexity

To some extent, interface complexity is a product of mathematical complexity, because requiring users to enter more input parameters requires a more detailed interface. But there is a difference between simplicity and clarity. Complex interfaces can be unclear unnecessarily. LiST and other models should strive for interface clarity, including improved graphic design, choosing which inputs are hidden or exposed by default (with or without default data). This might also mean alternative layout flows, for example, guided walk-though “wizards” to aid user interaction and comprehension. The LiST team recently recruited specialists in interface design to work with them on exploring these ideas.

#### Interface variants for particular use cases

LiST can be used for various purposes (evaluation, decision-support, advocacy) [9] and interventions (maternal, child, curative, preventative, diarrhea, malaria) [7]. Sometimes users only want or need to use certain aspects of LiST. One way to maintain accuracy while increasing usability might be to develop interface variants, or stand-alone applications, that focus on isolated components of LiST for specific purposes. For example, an interface for nutrition applications might only expose the inputs to the user that he or she is likely to have available, and hide other parts of the model. An interface for advocacy might only give a set of results that a user can immediately take and use in reports and documents. A full version of the software could continue to be built out with features such as uncertainty, and custom interventions. Such variants might make it easier for policy makers in particular spheres to engage with and run the tool. The LiST team has begun initial work on a nutrition-focused interface as a first example of such a variant.

#### More accessible tool-delivery mechanisms

There are aspects of usability that are not tied to the model itself or even the interface, such as the computer operating system and specifications required to run the software. Users in some contexts may require offline capabilities, but a web-based version could open up more options for enhanced usability. The LiST team, in partnership with the broader group that develops Spectrum, are working on an online version of LiST. Such an online version could allow for referencing up-to-date sources, immediate version control (always using latest version of the model), and shared user accounts. For some users and use cases, a mobile version of LiST might also be appreciated. For advanced users, LiST functions could be made available within a statistical package such as R, which would enable statisticians to incorporate LiST calculations as part of broader functions and models.

#### Expanded documentation and explicit justification for assumptions

Arguably LiST does a good job of allowing for both generic projections and complex, custom projections. The problem our respondents identified was that some users do not understand the options available to them and the implications of their choices. LiST has built-in documentation, but this could continue to be developed, with more immediate and clear explanations for the default assumptions that have been made. This could include simple, summary descriptions for those with limited time and skills (e.g. how to correctly interpret results), and more lengthy descriptions for those who want to understand the details (e.g. scientific justification and explanation of mathematical calculations).

#### Training resources for self-directed users

If LiST is to achieve greater uptake there needs to be a way for self-directed users to pick up the tool themselves and start working with it independently. If users must always attend a training session, this will inevitably limit uptake. There needs to be a way for potential users who cannot attend trainings, or who have attended trainings in the past, to start (again) themselves. With this in mind, the LiST team continues to invest in training resources, and online webinars, to help self-directed users run both simple and complicated projections.

#### Advocating for continued science and data collection

LiST needs flexibility and customizability where it is wanted, and the best possible default data and assumptions where it is not. The LiST team already makes great efforts to maintain default data, including coverage and cause-of-death data for 157 countries. LiST also leads scientific efforts to obtain reliable effectiveness values [7, 10]. Although LiST could take even greater responsibility to curate a set of reliable default data (e.g., regional or district models, or alternative demographic projections), this is arguably beyond LiST’s mandate and in any case would require a big investment of resources. Ultimately, for specific projections, users may need to bring their own data to the table. LiST could be clearer about what is required, how to get it, and the implications of using generic or low-quality data. Advocating for data responsibility in this way would not only improve the accuracy of LiST results, but would foster greater awareness among users, and the wider international community, of the need for and value of high-quality data.

## Conclusions

Organizations in all sectors are placing increasing emphasis on data for decision making. To achieve the gains of this data revolution in public health, government and agency staff at *all levels* should be empowered to use the data available to them, including the use of models to make full use of the information they can generate. For this, we need tools that meet a threshold of both accuracy, so results clarify rather than mislead, and usability, so tools can be used readily and widely, not just by select experts. Although for some users LiST already meets these standards, there are ways in which LiST might continue to be improved or adapted to further advance its uptake and impact.

## Additional file

Additional file 1: Qualitative survey. (PDF 33 kb)
